# Adsorptive Behavior and Voltammetric Determination of Hydralazine Hydrochloride at A Glassy Carbon Electrode Modified with Multiwalled Carbon Nanotubes

**Published:** 2017

**Authors:** Mehdi Khodadadian, Ronak Jalili, Mohammad Taher Bahrami, Gholamreza Bahrami

**Affiliations:** a *Medical Biology Research Center, Kermanshah University of Medical Sciences, Kermanshah, Iran. *; b *School of Pharmacy, Kermanshah University of Medical Sciences, Kermanshah, Iran.*

**Keywords:** Carbon nanotube, Hydralazine, Modified electrode, Adsorption, Stripping voltammetry

## Abstract

An electroanalytical method has been introduced for highly sensitive determination of hydralazine hydrochloride (Hy-HCl) based on its oxidation at a glassy carbon electrode modified with multiwalled carbon nanotubes (MWCNT/GCE). Studies showed that the electrochemical oxidation of Hy-HCl was accompanied by adsorption and highly sensitive responses could be achieved by adsorptive stripping voltammetry. The electrooxidation of Hy-HCl at MWCNT/GCE occurred at ~32 mV which was lower than that observed at bare GCE (~52 mV). The optimum working conditions for determination of the drug using differential-pulse adsorptive stripping voltammetry (DPAdSV) were established. The method exhibited linear responses to Hy-HCl in the concentration range 10-220 nM with a detection limit of 2.7 nM. The proposed method was successfully applied to the determination of this compound in pharmaceutical dosage forms.

## Introduction

**Scheme 1:**
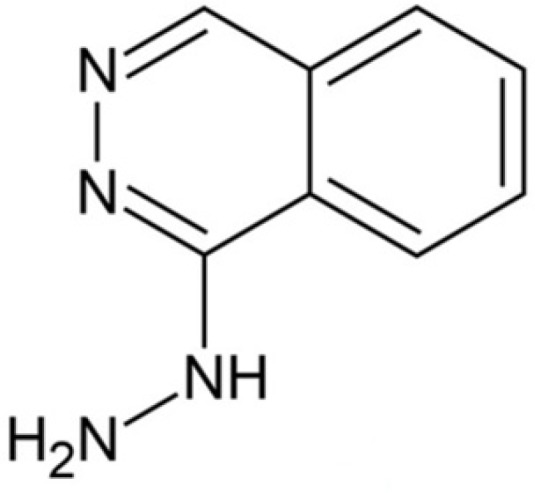
Chemical structure of hydralazine

Hydralazine (1-hydrazinylphthalazine) (Scheme 1) is a direct-acting smooth muscle relaxant and has experienced extensive clinical application in the treatment of both hypertension and chronic heart failure (1, 2). Several techniques including colorimetery (3), spectrophotometry (4), fluorimetry (5), chemiluminescence (6), gas chromatography (7), high performance liquid chromatography (8, 9) and electrochemistry (10, 11) have been used to analyze Hy-HCl in various samples.

Electroanalytical techniques have some important advantages including speed, high sensitivity, relative simplicity and low costs compared to other techniques. Adsorptive stripping voltammetry has been demonstrated to be a highly sensitive electroanalytical method for the determination of a wide range of electroactive compounds (12-15). The method utilizes controlled interfacial accumulation of the analyte on to the electrode surface as an effective preconcentration step prior to voltammetric determination. 

In recent years, chemically modified electrodes have received a great deal of attention due to their potential applications in electroanalytical chemistry (16-18). Modification of electrode’s surface with various modifiers such as metal nano-particles (19), carbon nanotubes (20), graphene (17), transition metal complexes (21), ionic liquids (22), enzymes (23), polymers (24), and various nano-composites (25, 26) have been reported. It has been observed that the modification of electrode’s surface with multiwalled carbon nanotubes (MWCNTs) would result in low detection limits, reduced overpotentials and resistance to surface fouling and therefore MWCNTs have been claimed as electrocatalysts (27). Due to their unique properties, these materials offer enormous possibilities in the field of pharmaceutical analysis. Some interesting applications include the voltammetric determination of cefpirome in bulk form and pharmaceutical formulation at multiwalled carbon nanotube modified glassy carbon electrode (28), the simultaneous determination of cysteamine and folic acid in pharmaceutical and biological samples using multiwall carbon nanotube modified paste electrode (29), the highly sensitive voltammetric sensor for qualitative and quantitative determination of phenobarbital as an antiepileptic drug in presence of acetaminophen (30), and also the sensitive voltammetric sensor for determination of synthetic corticosteroid triamcinolone, abused for doping (31).

To our knowledge, there is no previous report on the electrochemical behavior and determination of Hy-HCl at electrodes modified with MWCNTs. The strong adsorption of the drug at the surface of MWCNT/GCE enabled us to reach highly sensitive measurements. The proposed method was successfully applied to the determination of Hy-HCl in pharmaceutical formulations. 

## Experimental

*Materials*

Hy-HCl was obtained from Sigma-Aldrich (Madrid, Spain). MWCNTs with 95% purity (30-50 nm diameters and 3 μm length) were obtained from Timesnano Co. Ltd. (China). Stock solutions of Hy-HCl (1.0 × 10^−4^ M) was prepared in water and kept in darkness at 4 °C. Phosphate buffer solutions (PBS, 0.1 M) were prepared by mixing appropriate amounts of Na_2_HPO_4_ and NaH_2_PO_4_ in distilled water, and then adjusted to the desired pH value by NaOH and H_3_PO_4_ solutions. Working solutions were freshly prepared by diluting the stock solution with 0.1 M PBS. Other chemicals were of analytical grade and used without further purification.

**Scheme 2:**
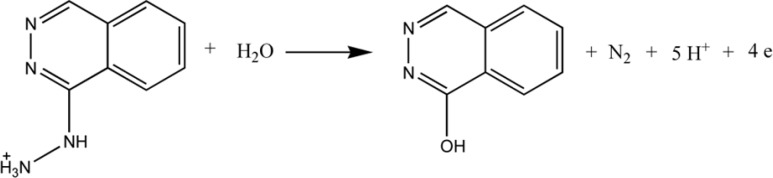
Proposed electrode reaction of Hy-HCl

**Figure 1:**
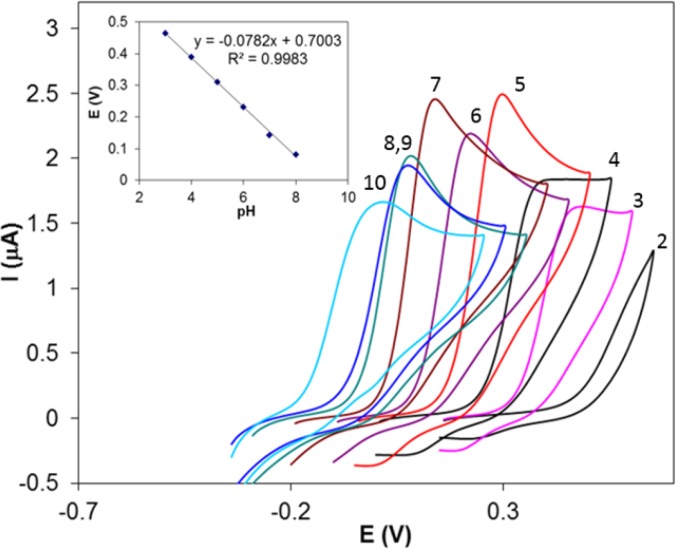
Cyclic voltammograms of 0.65 µM Hy-HCl in 0.1 M PBS at different pH values. Inset shows potential *vs.* pH curve

**Figure 2:**
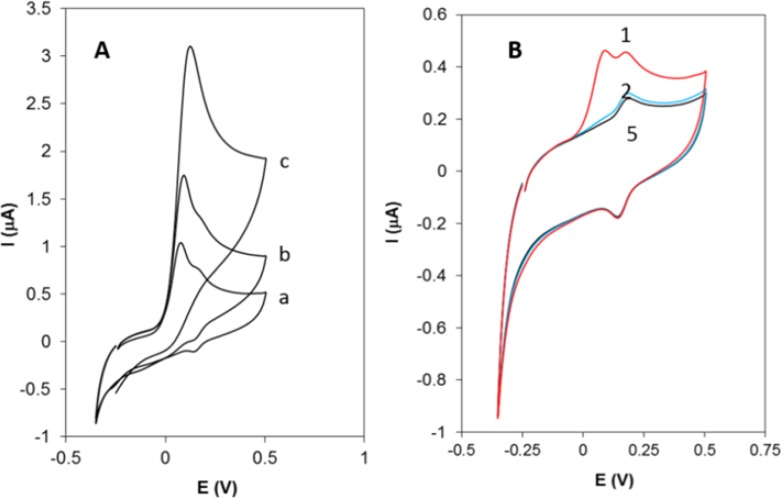
A) Cyclic voltammograms of Hy-HCl at GCE in 0.1 M PBS (pH 7.0) with different concentrations: (a) 0.2, (b) 0.4 and (c) 0.8 µM. (B). Successive cyclic voltammograms recorded at a GCE in 0.1 PBS solution already dipped in 0.2 µM solution of Hy-HCl for 60 sec

**Figure 3:**
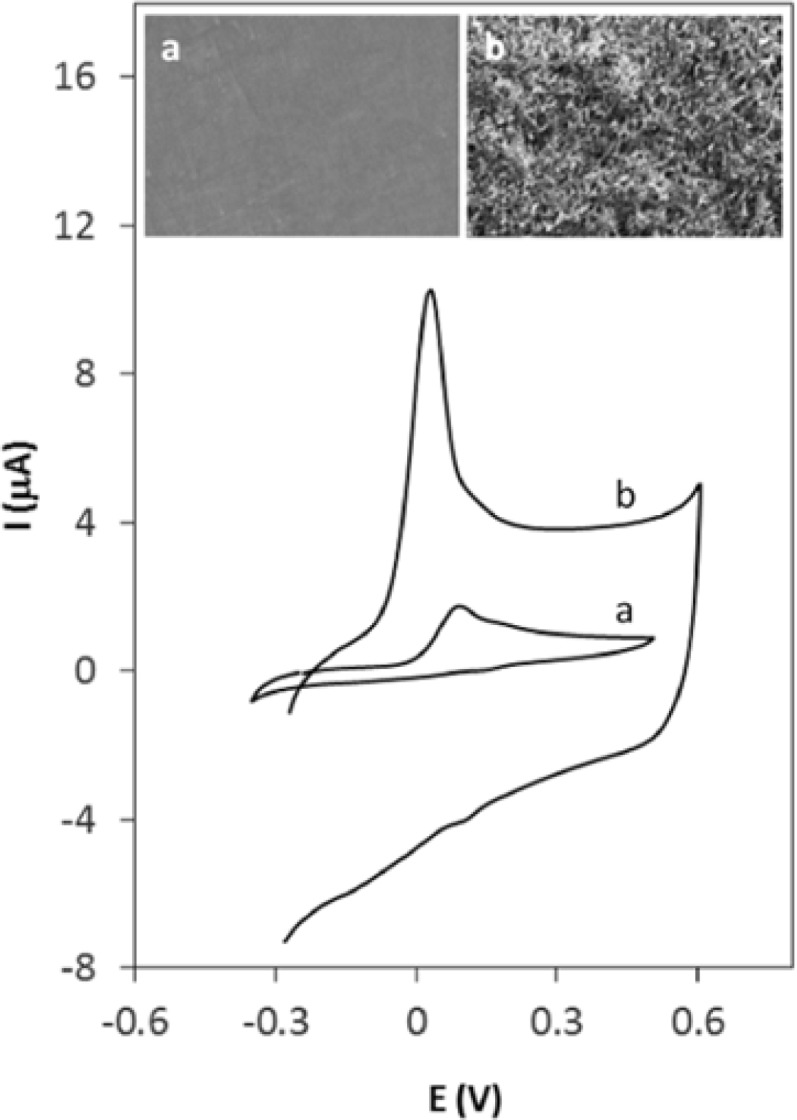
Cyclic voltammograms of 0.4 µM Hy-HCl in 0.1 M PBS (pH 7.0) at (a) GCE and (b) MWCNT/GCE with 60 sec accumulation time

**Figure 4:**
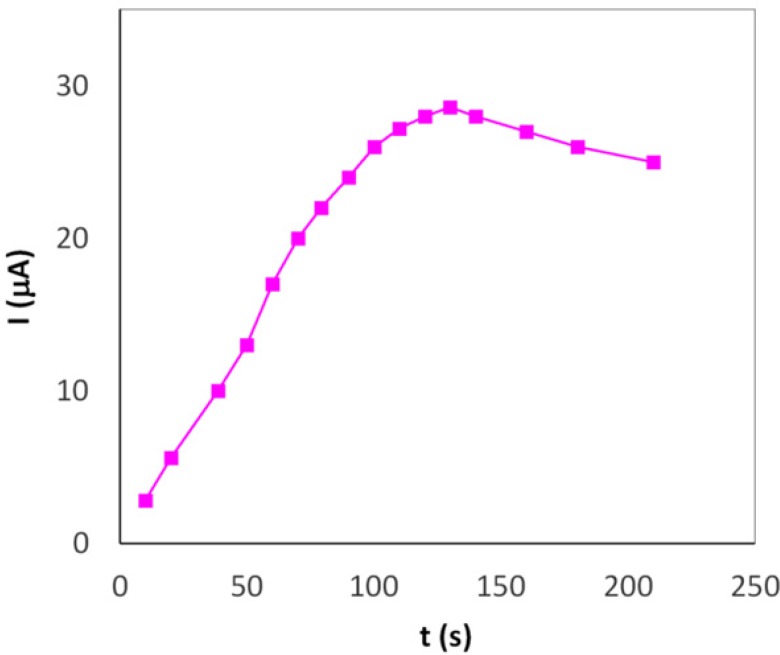
Effect of accumulation time on the oxidation peak currents of 0.22 µM Hy-HCl (0.1 M PBS, pH 7.0) in open circuit potential

**Figure 5:**
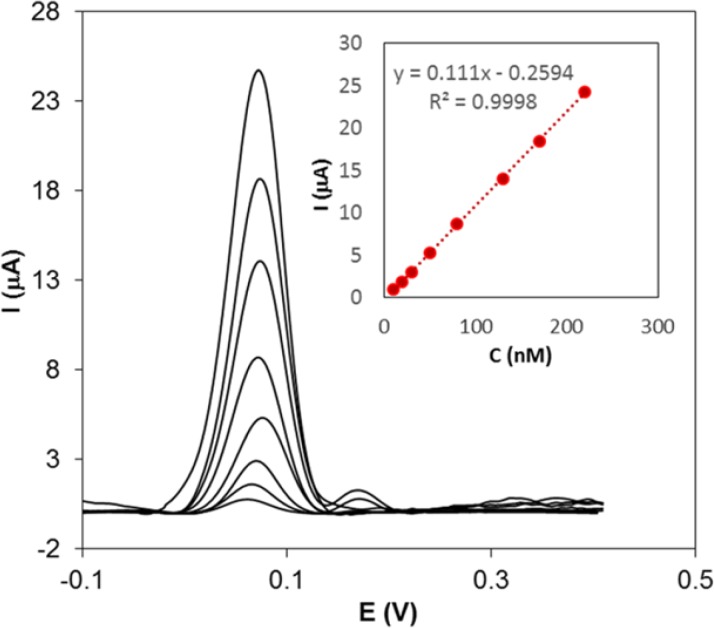
DPVs for a solution containing different concentrations of Hy-HCl (10-220 nM) at MWCNT/GC electrode. Scan rate: 50 mVs^−1^, accumulation time: 120 sec, electrolyte: 0.1 M PBS (pH 7.0). Inset plot shows the linear dependence of current *vs.* concentration

**Figure 6:**
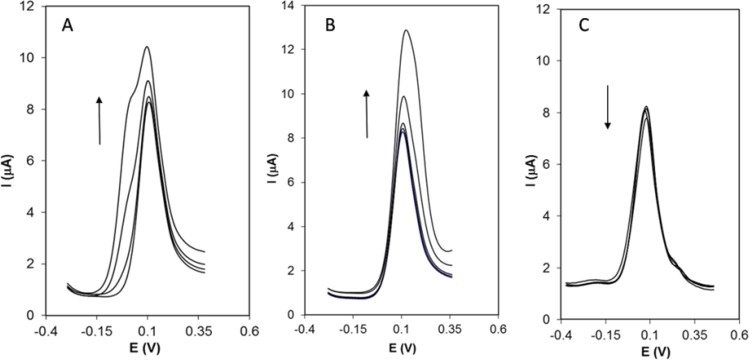
DPVs of 50 nM Hy-HCl in the presence of A) 0.0, 5, 20 and 50 μM ascorbic acid:, B) 0.0, 0.05, 0.1, 0.3 and 0.9 μM dopamine, C) 0.0, 10, 50 and 100 μM urea

**Table 1:** Determination of Hy-HCl in tablets

**Sample**	**Amount labeled** **(mg)**	**Amount found** **(mg)**	**RSD (%)** **n = 5**	**Recovery** **(%)**
1	10.0	10.13	3.1	101.3
2	25.0	24.63	2.5	98.5
3	50.0	51.02	1.9	102.0

*Instrumentation*

Voltammetric measurements were carried out with an AUTOLAB (Eco Chemie B. V.) PGSTAT30 potentiostat/galvanostat. The electrochemical cell was assembled with a saturated Ag/AgCl reference electrode, a Pt wire counter electrode, and the prepared working electrodes. All electrochemical measurements were carried out at room temperature. The surface morphology of modified electrodes was characterized with a scanning electron microscope (SEM) (KYKY-EM3200).

*Preparation of working electrodes*

Glassy carbon electrodes were polished to a mirror-like surface with 1.0 and 0.3 µm alumina slurry, and then sonicated in water and absolute ethanol, respectively. MWCNT/GCE was prepared by casting 10 μL of MWCNT-ethanol dispersion (0.5 mg mL^−1^) on the GC electrode surface and dried in an oven at 50 ºC to get a film covering the electrode surface (noted as MWCNT/GCE). Finally, the electrode was rinsed with double distilled water before measurements.

*General procedure for recording voltammograms*

The general procedure for obtaining voltammetric curves was as follows: 10.0 mL of 0.1 M phosphate buffer (pH 7.0) was transferred into the voltammetric cell and its voltammogram was recorded over the potential range -0.2 to 0.5 V on a MWCNT/GC electrode following 120 sec accumulation time in open circuit potential. This voltammogram was considered as background signal. The required aliquot of the standard solution of Hy-HCl was added by means of a micropipette and its voltammogram was recorded as before. The pulse amplitude of 100 mV, pulse width of 250 ms and a scan rate of 50 mV s^−1^ were used for differential pulse voltammetry (DPV). All experiments were carried out at ambient laboratory temperature (25 °C). 

*Sample Preparation*

The analyzed samples were different batches of hydralazine tablets (Kwality Pharmaceuticals Pvt. Ltd.). Five tablets were grounded, mixed and homogenized. An amount equivalent to one tablet was accurately weighed and dissolved in 50 mL of distilled water with the aid of ultrasonication for 2 min. After sonication, the suspension was allowed to settle down and more diluted solutions were prepared from the clear supernatant so that the final concentration of the drug fell within the calibration range of the voltammetric technique. The last solution was prepared in 10 mL of 0.1 M PBS (pH 7.0) and transferred into the electrochemical cell for voltammetric analysis. 

*Controlled potential coulometry*

Using controlled potential coulometry, the number of electrons transferred, *n, *were found out from the charge consumed by 25 mL of 1 × 10^−6^ M Hy-HCl. The charge consumed for the electrolysis was found as 9.91 × 10^−3^ *C*. The coulometric *n *was calculated using the Equation *Q *= *nFN*, where *Q *is charge in coulombs, *F *is faraday’s constant and *N *is number of moles of the substrate. The *n *value is found to be four (rounded value) for anodic peak of Hy-HCl. 

## Results and Discussion

*Electrochemical behavior of Hy-HCl*

The electrochemical behavior of Hy-HCl was first studied at bare GCE and at different pH values. Cyclic voltammetry studies in aqueous solutions showed that the electrode reaction of Hy-HCl was an irreversible oxidation process and good sensitivities could be achieved within neutral pH values (Figure 1). Increasing pH of the solution shifted the peak potentials toward less positive values. The plot of peak potential versus solution pH between 2.0 and 8.0 showed a straight line expressed by the Equation y = -0.078x + 0.700 (R^2 ^> 0.99). Considering the electrochemical oxidation of hydrazine compounds a 5H^+^/4e mechanism is expected for Hy-HCl (32). The proposed electrooxidation mechanism for this compound is depicted in Scheme 2. 

Figure 2A shows the cyclic voltammograms of Hy-HCl at bare GCE at different concentrations. As seen, the shape of CV is concentration dependent. At lower concentrations the CV shows a “post wave” which might be either due to the strong adsorption of the reactant at the electrode surface (33) or formation of some electroactive products. At higher concentrations, however, these small waves are not observable. Figure 2B displays successive cyclic voltammograms recorded at a GCE in 0.1 M PBS solution already dipped in a 0.2 µM solution of Hy-HCl for 60 sec. The first cycle shows the post waves following an irreversible wave at 0.1 V. The peak at 0.1 V reflects the weakly adsorbed analyte on the electrode surface since it completely disappeared after the first cycle. The pair at 0.142 and 0.162 V, however, was still observable and stable after five scans and varied linearly with scan rate (v) (R^2 ^> 0.99), which implies a surface confined process. Our preliminary studies revealed that Hy-HCl could be efficiently accumulated on MWCNT modified electrodes. Therefore, experiments with GCE and electrodes modified with MWCNT were carried out in order to probe the electrochemical behavior of Hy-HCl on these electrodes. In all experiments, the cyclic voltammograms were recorded with electrodes submerged in a PB solution containing 0.4 µM Hy-HCl adjusted to pH 7.0 and an accumulation time of 60 sec. As shown (Figure 3), a weak anodic peak corresponding to the oxidation of Hy-HCl is observed on the GC electrode (curve a), while on the MWCNT/GCE the peak current increased substantially (curve b). Clearly, modification of glassy carbon electrode with MWCNTs leads to a strong accumulation of Hy-HCl, which provides a preconcentration step for highly sensitive adsorptive stripping measurements. Also, the oxidation of Hy-HCl at MWCNT occurred at a lower potential (~32 mV) than that observed at bare GCE (~52 mV). The excellent properties of MWCNT, such as its high electrical conductivity and high surface area may also contribute to the observed electrochemical response. 

The scanning electron micrographs (insets of Figure 3) clearly shows that the MWCNTs are distributed uniformly on the surface glassy carbon (b). The diameter of the nanotube deposits was found to be about 30 to 50 nm. The spaghetti-like MWCNTs formed a porous structure leading to an increased surface area and hence an enhanced electrochemical response.

*Effects of accumulation potential and time*

The oxidation peak current of Hy-HCl (0.22 µM) was measured by cyclic voltammetry after 60 sec accumulation time at different potential values from -0.2 to 0.5 V in 0.1 PBS (pH 7.0). It was observed (data not shown) that the oxidation peak current of Hy-HCl was remained unchanged up to 0.1 V and thereafter decreased rapidly with accumulation potential. Our results, however, showed that the drug can be effectively accumulated on MWCNT surface without any applied potential. The preconcentration, therefore, was performed under open-circuit potential. Figure 4 shows the influence of accumulation time on the oxidation peak current of 0.22 µM Hy-HCl. The current was increased greatly at first, and then decreased after 140 sec due to the adsorption saturation. An accumulation time of 120 sec was chosen for subsequent experiments.

*Adsorptive stripping voltammetric determination of Hy-HCl*

To estimate the lower detection limit and the linear calibration range of Hy-HCl, DPV method was used. The DPVs were recorded by changing the concentration of Hy-HCl. As can be seen from Figure 5, an anodic peak at 70 mV was appeared. The analytical plot was linear in the concentration range of 10-220 nM (R^2 ^= 0.999) Hy-HCl. The limit of detection (LOD) were calculated from the Equation LOD = 3 s/m; where s represents the standard deviation of the signal of the blank (n = 6) and m represents the slope of the calibration curve. 

The LOD of Hy-HCl was found to be 2.7 nM. The repeatability was determined by successive measurements (n = 5) of a 50 nM Hy-HCl solution and relative standard deviations of 1.81% was obtained. These results clearly indicate that the proposed electrode can be utilized for the sensitive and precise determination of this compound. 

*Interference studies*

The effect of some interfering substances was investigated by adding the compounds to a solution containing 50 nM Hy-HCl in 0.1 M PBS (pH 7.0). The tolerance limit was taken as the maximum concentration of the foreign substances, which caused an approximately ±5% relative error in the determination of the analyte. Common ions such as Na^+^, K^+^, Cl^-^, CO_3_^2-^, PO_4_^3-^, and SO_4_^2-^ as well as starch did not show any interference with Hy-HCl detection. 

The results showed that 800-fold saccharin, glucose, sucrose, urea; 600-fold glycine, phenylalanine, lysine, glutathione, citric acid, 350-fold ascorbic acid, uric acid, Cu^2+^, and 2-fold dopamine are tolerable in voltammetric determination of Hy-HCl. Figure 6 represents DPVs of Hy-HCl in the presence of increasing concentrations of some interfering species.

*Analytical application*

The proposed electrode was used to analyze Hy-HCl in pharmaceutical formulations (Table 1). The results were satisfactory with the recoveries ranging from 98.5 to 102.0%. From these results, it can be concluded that the MWCNT/GCE shows good performance for the routine analysis of this compound in pharmaceutical formulations.

## Conclusions

The results discussed above demonstrate that the electrochemical response of Hy-HCl by adsorptive stripping voltammetry on MWCNT/GCE can be markedly enhanced within neutral pH values. The presence of MWCNT on the electrode surface not only boosted the current response, but also shifted the oxidation potential to lower values. The reliability and fast analytical determination of Hy-HCl on MWCNT/GCE could be applied to the routine analysis of this compound in pharmaceutical samples. 
